# Antioxidant Constituents of *Cotoneaster melanocarpus* Lodd

**DOI:** 10.3390/antiox2040265

**Published:** 2013-10-24

**Authors:** Veronika M. D. Holzer, Agnieszka D. Lower-Nedza, Myagmar Nandintsetseg, Javzan Batkhuu, Adelheid H. Brantner

**Affiliations:** 1 Department of Pharmacognosy, Institute of Pharmaceutical Sciences, Karl-Franzens University, Universitaetsplatz 4/I, Graz 8010, Austria; E-Mails: veronika.holzer@edu.uni-graz.at (V.M.D.H.); agnieszka.ln86@gmail.com (A.D.L.-N.); 2 Laboratory of Bioorganic Chemistry and Pharmacognosy, Department of Biochemistry and Bioorganic Chemistry, School of Biology and Biotechnology, National University of Mongolia, P.O.B-617, Ulaanbaatar-46A, Mongolia; E-Mails: nandia0520@yahoo.com (M.N.); jbatkhuu@num.edu.mn (J.B.)

**Keywords:** *Cotoneaster melanocarpus*, DPPH, antioxidants, TLC, HPLC, LC-MS

## Abstract

The aim of this study was the evaluation of the antioxidant capacity of *Cotoneaster melanocarpus* Lodd. and the identification of antioxidant active constituents of this plant. *C. melanocarpus* Lodd. is a shrub indigenous to Mongolia and used in Traditional Mongolian Medicine as a styptic. Before extraction, the plant material was separated into three parts: young sterile shoots, older stems and leaves. All these parts were extracted with water, methanol, ethyl acetate, dichloromethane and hexane, successively. The methanolic extract of the sterile shoots showed the highest antioxidant activity in the DPPH (2,2-diphenyl-1-picrylhydrazyl) radical scavenging assay (IC_50_ 30.91 ± 2.97 µg/mL). This active extract was further analyzed with chromatographic methods. TLC fingerprinting and HPLC indicated the presence of the flavonol glycosides quercetin-3-*O*-rutinoside (rutin), quercetin-3-*O*-galactoside (hyperoside) and quercetin-3-*O*-glucoside (isoquercetin), ursolic acid as well as chlorogenic acid, neochlorogenic acid and cryptochlorogenic acid. The findings were substantiated with LC-MS. All identified compounds have antioxidant properties and therefore contribute to the radical scavenging activity of the whole plant.

## 1. Introduction

The process of ageing is very complex and universal to every organism. It can be characterized as a progressive deterioration in physiological functions and metabolic processes, ultimately leading to sickness and death [1]. It is well established that reactive oxygen species (ROS) and other free radicals play a significant part in the degeneration of cell tissue. Many ROS are by-products of the normal human metabolism and play an important part in cell signaling and the immune response [2,3]. However, they also can cause oxidative damage to macromolecules leading to a variety of age-related disorders and other diseases. An uncontrolled rise in ROS, referred to as oxidative stress, is known to contribute to diabetes mellitus, rheumatoid arthritis, atherosclerosis, essential hypertension, cancer and neurodegenerative diseases such as Parkinson’s and Alzheimer’s among others [1,4,5].

Plant antioxidants are part of our daily nutrition and many medicinal plants are proven to have a high antioxidant capacity [6]. These external antioxidants work together with internal antioxidant defense mechanisms to inhibit oxidation processes in the human body [7]. The *Cotoneaster* genus belongs to the *Rosaceae* family and is widely distributed around the globe. Although they are mostly planted for ornamental purposes, various species are used to treat different disorders in traditional medical systems throughout Asia [8,9]. *C. melanocarpus* Lodd. is a shrub native to Mongolia and a frequent species of the shrub layer in many subtaiga forests of the area [10]. Its fruit and shoots are used in Traditional Mongolian as well as in Traditional Tibetan Medicine as a styptic to treat nasal haemorrhage, excessive menstruation and hematemesis [11]. Despite the fact that it is used as a medicine, not much is known about its chemical composition.

In a previous screening of Mongolian medicinal plants at the National University of Mongolia a certain antioxidant activity was found for methanolic extracts of the stems of *C. melanocarpus* [12]. On the basis of this antioxidant activity and due to the absence of studies that would indicate the plant metabolites contributing to this activity, this study was performed to identify some of the main antioxidant constituents.

## 2. Experimental Section

### 2.1. Plant Material and Extraction

The plant material was collected in Mongolia in August 2011, Tov aimag, Bayannchandmani sum, in the forest east of the Chandmanbulag tourist camp. It was identified by Professor Ts. Jamsran of the School of Biology and Biotechnology of the National University of Mongolia*.* A voucher specimen (No. 48.04.0310A) is deposited at the herbarium of the Laboratory of Bioorganic Chemistry and Pharmacognosy, School of Biology and Biotechnology, National University of Mongolia.

The plant material was separated into three parts: young sterile shoots, older stems and leaves. Young sterile shoots were defined as non flowering shoots growing from the ground which were less than one year old and not branched. All parts were dried in the shade. For the extraction, each part was first extracted with water (H_2_O) in the ratio 1:10 at boiling temperature for 30 min. The filtrate was freeze dried and the filtration residue air-dried under a fume hood. The dry filtration residue was then extracted with methanol in the same fashion and subsequently with ethyl acetate (EtAc), dichloromethane (DCM) and hexane in this order. The extracts were concentrated with a rotary evaporator and afterwards air-dried, the methanol extract was vacuum dried. The extracts were stored at 4 °C.

### 2.2. DPPH Radical Scavenging Assay

To analyze the antioxidant ability of the extracts, a modified version of the DPPH (Sigma) radical scavenging method described by Mensor *et al*. [13] was applied. On 96-well microtiter plates (Sterilin), 75 µL of a 0.3 mM DPPH solution in methanol and 75 µL of the extracts (400, 200, 100, 50, 25 for and 12.5 µg/mL in methanol) were mixed and incubated for 30 min in the dark. The absorption was measured at 517 nm against a blank (75 µL sample mixed with 75 µL methanol) in a microplate reader (Perkin Elmer Wallac Victor2 1420 Multilable Counter). Furthermore, a standard for DPPH (75 µL DPPH solution and 75 µL methanol) was measured against methanol. Rutin was used as a reference. The percentage of the radical scavenging activity was calculated by means of the following equation:

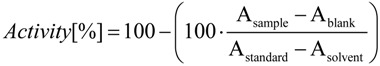
(1)
*A*_sample_ is the absorption value of the sample at a given concentration, *A*_blank_ the absorption value of the respective blank, *A*_standard_ the absorption value of the DPPH standard and *A*_solvent_ the absorption value of methanol.

The test was done in triplicate and repeated 6 times on different days for validation. With the calculation of a standard curve the IC_50_ value for each extract was determined.

### 2.3. TLC Profiles of the Extracts

TLC fingerprints were made of all extracts. As references rutin, hyperoside, chlorogenic and ursolic acid were used. Aqueous and methanolic extracts were evaluated in a flavonoid TLC system [14]. One hundred µg of extracts were applied onto Merck Silica gel 60 F254 plates and chromatographed with ethyl acetate:formic acid:acetic acid:water 100:11:11:27. The plate was then sprayed with natural products reagent (1% methanolicdiphenylboric acid-ethylamino ester) followed by 5% ethanolic polyethylene glycol-4000 reagent. The plate was viewed under 366 nm. Methanolic, ethyl acetate and dichloromethane extracts were chromatographed with toluene:diethyl ether 1:1 (saturated with 10% acetic acid) and detected with anisaldehyde reagent at 366 nm. Dichloromethane and hexane extracts were separated with toluene:ethylacetate:acetic acid 93:6:1 and also detected with anisaldehyde reagent at 366 nm.

### 2.4. Analytical HPLC Method

The analysis for the methanolic extract of the young sterile shoots was carried out on a LiChroCART (250-4 RP-18, 5 mm) column with an Agilent Technologies 1100 Series HPLC system equipped with a photo-diode array detector. The chromatographic separation was achieved with a gradient of two solvents (A 0.3 v/v % phosphoric acid in water, B acetonitrile) listed in Table 1. The flow rate was 1 mL/min, the injection volume 10 µL (sample concentration 5 mg/mL, reference concentration 1 mg/mL). Peaks were detected at 370, 320, 270 and 210 nm. The HPLC fingerprint was performed at 270 and 210 nm because most of the compounds could be detected under these conditions. The retention times for the pure reference substances are as follows: chlorogenic acid 7.64 min, rutin 19.05 min, hyperoside 19.33 min, isoquercetin 19.96 min, ursolic acid 44.25 min.

**Table 1:** Solvent gradient for HPLC analysis.

Time (min)	Solvent A ^a^ [%]	Solvent B ^b^ [%]
Initial	90	10
20	80	20
30	60	40
40	10	90
50	0	100
55	0	100

^a^ 0.3 v/v % phosphoric acid in water; ^b^ acetonitrile.

### 2.5. LC-MS Analysis

For confirmation of the constituents, the HPLC system was adapted to be fit for LC-MS. The same gradient (Table 1) was used but Solvent A was changed to 0.1 v/v % formic acid in water and Solvent B to 0.1% formic acid in acetonitrile. The analysis was carried out on an LC-MS system (Ultimate 3000 RS coupled with LCQ Deca XP Thermo Scientific), and a different column (Phenomenex Synergi 2.5 µm Fusion-RP 100 A 100 × 2.0 mm) was used. The flow rate was set at 0.25 mL/min and the injection volume at 10 µL (sample concentration 5 mg/mL, reference concentration 1 mg/mL). The ions were produces by electro spray in negative mode with a collision energy of 30%, an ionization voltage of 3 kV, a capillary temperature of 330 °C, a sheath gas flow rate of 50 arbitrary units, and an auxiliary gas flow rate of 9 arbitrary units.

## 3. Results and Discussion

### 3.1. DPPH Radical Scavenging Assay

The DPPH (2,2-diphenyl-1-picrylhydrazyl) radical scavenging assay is a very reliable way to determine the ability of plant extracts to scavenge free radicals. In this assay the decolourisation of the radical is measured photometrically after it reacts with substances that can donate hydrogen atoms [15]. The results of the DPPH radical scavenging assay are shown in Table 2. The methanol extract of the young sterile shoots proved to have the highest antioxidant effect whereas the less polar extracts showed considerably less to no activity. The methanol extract was therefore further analyzed to determine the main phenolic constituents.

**Table 2:** Results of the DPPH radical scavenging assay. Results are given as IC_50_ ± SD (µg/mL).

Extraction medium	Sterile shoots	Old stems	Leaves
H_2_O	53.53 ± 6.21	86.72 ± 7.50	67.74 ± 6.14
MeOH	30.91 ± 2.97	48.21 ± 4.38	106.41 ± 12.26
EtAc	124.07 ± 15.80	74.88 ± 14.59	>200
DCM	>200	134.60 ± 32.77	>200
Hexane	no activity	no activity	no activity
Reference rutin	13.26 ± 0.58		

### 3.2. TLC Profiling

**Figure 1:**
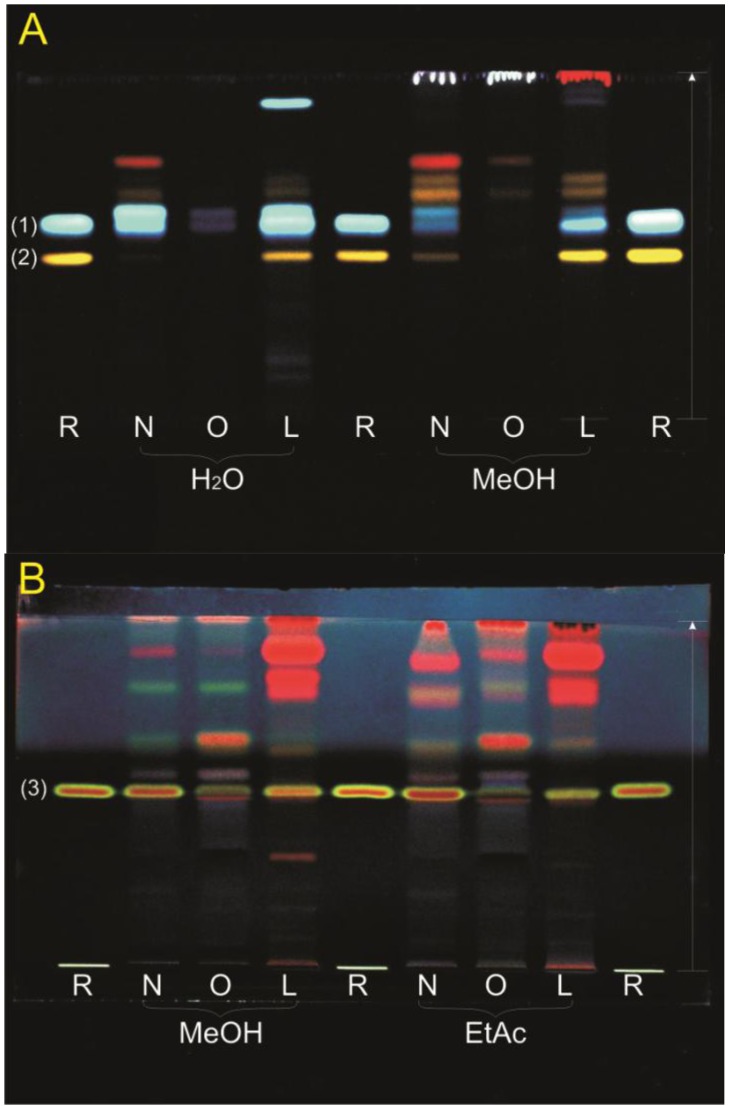
TLC profiles of the extracts. Extracts: (N) sterile shoots, (O) older stems, (L) leaves; References R: (1) chlorogenic acid *R*_f_ = 0.55, (2) rutin *R*_f_ = 0.46, (3) ursolic acid *R*_f_ = 0.50; Mobile phase and detection: (**A**) ethyl acetate:formic acid:acetic acid:water 100:11:11:27, natural products reagent and PEG, 366 nm; (**B**) toluene:diethyl ether 1:1 (saturated with 10% acetic acid), anisaldehyde reagent, 366 nm.

TLC profiles were made of all extracts. The extracts were investigated with different TLC systems to separate the compounds and to investigate constituents with suitable pure reference substances. Many substances were tested as references, only four showed similar migration distances as compounds in the extract. For the polar extracts emphasis was set on flavonoids which presented with an orange fluorescence in the TLC after derivatization with natural products reagent and PEG. The flavonol glycosides quercetin-3-*O*-rutinoside (rutin), quercetin-3-*O*-galactoside (hyperoside) and quercetin-3-*O*-glucoside (isoquercetin) had similar *R*_f_-values when compared to the *R*_f_-values of compounds native to the aqueous and methanolic extracts. Rutin seemed to be present especially in the leaves and to a lesser extent in the sterile shoots. It was not visible in the extracts of the old stems. Chlorogenic acid was present mostly in the polar extracts of the leaves and young sterile shoots (Figure 1A). In all extracts except the aqueous extracts, the characteristic fluorescence of ursolic acid when sprayed with anisaldehyde reagent (red-brown with lime-green border) was discovered (Figure 1B).

### 3.3. HPLC and LC-MS Analysis

**Figure 2:**
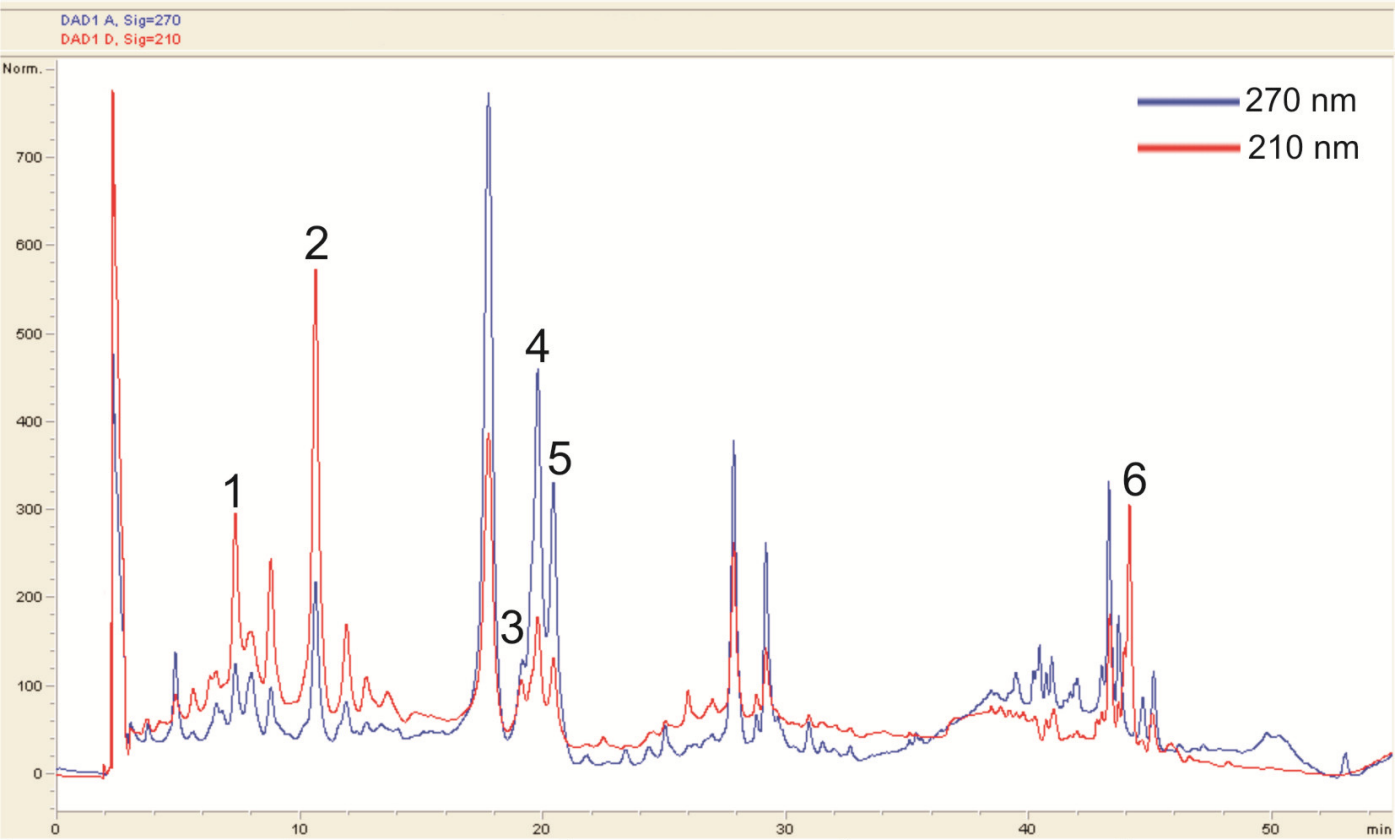
HPLC fingerprint of the methanol extract of the young sterile shoots.

Because the methanol extract of the young sterile shoots presented the highest radical scavenging ability it was selected for further chromagraphic studies. Therefore, an HPLC chromatographic separation was performed. A typical chromatogram is depicted in Figure 2. The peak purity was confirmed with UV spectra obtained by photo-diode array. The peaks were identified by relating the retention time of pure substances to the peaks in the HPLC spectra, as well as the UV and the mass spectra obtained by LC-MS. For a list of peaks and identified compounds see Table 3.

**Table 3:** Identified compounds and their retention time (*R*_t_) in the HPLC fingerprint.

Peak No.	Compound	*R*_t_ [min]
1	Neochlorogenic acid	4.74
2	Chlorogenic acid	7.77
3	Rutin	19.04
4	Hyperoside	19.36
5	Isoquercetin	20.03
6	Ursolic acid	44.12

The HPLC analysis largely supported the findings of the TLC investigation. In most cases the HPLC system in use resulted in clearly separated peaks with distinct UV spectra. However, the identification of chlorogenic acid was complicated, because the UV spectrum obtained from the peak (Figure 2, peak 2) in the extract did not fully represent the spectrum of pure chlorogenic acid. The different absorption pattern indicated the presence of other substances. However, another peak (Figure 2, peak 1) strongly resembled the UV spectrum of chlorogenic acid, although the retention time was different. From the data gathered in the MS analysis, peak 1 was identified as neochlorogenic acid. Characteristic fragmentation patterns also indicated the presence of cryptochlorogenic acid. The MS identification of the isomers of chlorogenic acid matched the fragmentation pattern found by Clifford *et al*. [16].

No distinct rutin peak could be observed in the HPLC. However, its characteristic mass and fragmentation pattern were found in the MS investigation. Reference chromatograms and UV spectra suggested that the two distinct peaks at around 20 min were isoquercetin and hyperoside, respectively.

For ursolic acid, the detection wavelength was set at 210 nm. Because ursolic acid does not absorb light from 200 to 600 nm in a characteristic pattern, a co-chromatogram of ursolic acid and young shoot extract was made to confirm the presence of ursolic acid. No further peak appeared, which indicates that ursolic acid is native to the extract.

## 4. Conclusions

In summary, this study showed that the young sterile shoots of *C. melanocarpus* show an interesting antioxidant activity. The present investigation concluded that this antioxidant activity is based on the presence of polyphenolic compounds such as flavonoids as well as various plant acids. This plant has never before been investigated. Further investigations should be performed to elucidate the active principle in more detail.
